# “Everybody Wants to be Included”: Experiences with ‘Inclusive’ Strategies in Physical Education

**DOI:** 10.1007/s10882-022-09852-x

**Published:** 2022-06-22

**Authors:** Katherine Holland, Justin A. Haegele, Xihe Zhu, Jonna Bobzien

**Affiliations:** 1 Department of Teacher Education, Norwegian University of Science and Technology, Trondheim, Norway; 2 Department of Human Movement Sciences, Old Dominion University, Norfolk, Virginia USA; 3 Department of Communication Disorders and Special Education, Old Dominion University, Norfolk, Virginia USA

**Keywords:** Adapted physical education, Inclusion, Orthopedic impairment, Physical disability

## Abstract

This study examined how students with orthopedic impairments experienced strategies identified in the literature to support ‘inclusion’. An interpretative phenomenological analysis research approach was used, and six students with orthopedic impairments (age 10–14 years) served as participants. Data sources were written prompts, semi-structured, audiotaped interviews, and reflective interview notes. Based on thematic data analysis, four themes were constructed: “It’s kind of embarrassing”: experiences with support; “I don’t want to be different”: equipment, activity, and rule modifications; “I like to be a part of the conversation”: autonomy and choice in PE; and “I would rather be like the other students”: discussing disability. The experiences portrayed through these themes highlighted the differential effects of these explicated strategies, where each strategy contributed to feelings of inclusion, as well as marginalization among participants. As such, the findings indicated that ‘inclusive’ strategies should not be considered as blanket recommendations; instead, attempts to promote ‘inclusion’ of students with disabilities should start with a reflexive look at the unique needs of each individual student.

The Individuals with Disabilities Act (IDEA; 2004) mandates that all students, regardless of disability status, receive instruction in physical education (PE) as a part of a free and appropriate public education. For most students with disabilities, this PE instruction is provided in integrated PE settings alongside their peers without disabilities (Governmental Accountability Office [GAO], 2010; Heck & Block, 2020). Integrated settings, for the purposes of this article, are defined as placements or spaces in which students, regardless of unique educational needs, are educated together (Haegele, 2019). While movement toward the education of students with disabilities in integrated PE has become common internationally, it is not without concerns (Haegele, Wilson, et al., 2021). For example, in research examining how youth with orthopedic impairments experienced integrated physical education contexts, participants consistently reported being segregated or excluded from their peers without disabilities (Tanure Alves et al., 2020), instances of social isolation (Goodwin & Watkinson, 2000), experiences with incompetent or interfering help from peers (Goodwin, 2001), and a physical lack of access to PE settings (i.e., no ramp or lift to enter the gymnasium; Li & Chen 2012). As such, it is unsurprising that scholars encouraged more research and research-based practical strategies that might help to promote full, meaningful access to activity participation for individuals with disabilities in integrated PE settings (Haegele, Kirk, et al., 2020).

With the growth in the enrollment of students with disabilities in integrated PE contexts and concerns about the preparation of teachers to educate students with disabilities in their classes (Lieberman, et al., 2017), has come a proliferation of the explication of strategies for students with disabilities to experience ‘inclusion’ within these settings (Lieberman, et al., 2017; Williston, 2017). Specifically, stakeholders, such as K-12 teachers (Nagro et al., 2016; Williston 2017), teacher education faculty members, education researchers (e.g., Lieberman, et al., 2017), and parents (Wang, 2013), have recommended ‘inclusive’ strategies for practitioners to enhance educational opportunities. These strategies are often “simple, observable changes” (Haegele, Kirk et al., 2020, p. 10) intended to “foster inclusion and maximize student learning” (Lieberman, et al., 2017, p. 342). Some examples of explicated strategies include (a) having students with disabilities sit or stand in the same place as their peers during instruction (Lieberman, et al., 2019), (b) providing support from a peer-buddy (Wang, 2013), or (c) collaborating with students with disabilities on activity, rule, or equipment modifications (Lieberman, et al., 2017; Williston 2017). Since teachers are central to the quality of experiences of students with disabilities (Holland & Haegele, 2021), it has been posited that teachers can improve the quality of PE by implementing these types of ‘inclusive’ strategies (Lieberman, et al., 2019). Sixteen such strategies can be found in Table 1.

**Table 1:** ‘Inclusive’ Strategies for Integrated Classes

Topic	Definition
Activity Partners	The student with a disability should be partners with a peer during activities instead of an adult staff member.
Arrival/Departure	Ensure that the student with a disability arrives to and leaves from PE at the same time and with their peers without disabilities.
Autonomy*	Allow the student with a disability to have choice about the activities, equipment, and rules they engage with in PE.
Demonstrations	The student with the disability should be asked to demonstrate skills for the class as often as students without disabilities.
Differentiation	Differentiate instruction for students with disabilities, including but not limited to, providing additional demonstrations or alternate instructions.
Disability Discussion*	The teacher and student with a disability should have a discussion about the student’s disability and needs in their PE class.
Discussion with Peers*	The teacher should have a discussion with students without disabilities about ‘inclusion’ and how to treat the student with a disability.
Feedback	Students with disabilities should receive the same type and frequency of feedback as their peers without disabilities.
Fitness Testing	Students with disabilities should be assessed at the same time and in the same location as peers without disabilities.
Instruction	Ensure that the student with a disability is sitting or standing with their peers without disabilities when instructions are being given.
Modifications*	Provide modifications to rules, activities, or equipment for the student with a disability.
Non-Paraprofessional Adult*	Provide support from adapted PE teacher, occupational therapist, physical therapist, classroom special education teacher, or parent for the student with a disability.
Paraprofessional*	Provide hands-on paraprofessional support for the student with a disability.
Partner and Team Selections*	Partners and teams should be chosen by the teacher, not by the students.
Peer Buddy*	Assign a peer-buddy or peer-helper to the student with a disability.
Warm-Ups	Implement warm-ups for the entire class that are duration based rather than repetition based.

Note: *indicates strategies that evoked salient memories and were included in the analysis

To date, though, there is a paucity of research examining how students with disabilities experience PE contexts when these ‘inclusive’ strategies were implemented, and if these strategies helped foster feelings of inclusion. ‘Inclusion’ is a contentious term, that has been described as a “semantic chameleon” (Liasidou, 2012, p. 5) because it has been discussed in a variety of ways depending on the context in which it is used. For example, the PE literature has seen scholars utilize ‘inclusion’ to describe a physical space or placement analogous to integration (Qi et al., 2016; Reina et al., 2019), a philosophy related to the socially constructed environment within a PE class (Hutzler et al., 2005; Morley et al., 2005), as well as a subjective experience of participants within that space (Spencer-Cavaliere & Watkinson, 2010). As such, and consistent with recommendations from Graham & Slee (2008), it is important to identify our use of the term ‘inclusion’ to explicate our position and reveal the conceptualization that it is guiding our work. For the purposes of this study, Stainback and Stainback’s (1996) interpretation of inclusion as a subjective experience associated with feelings of belonging, acceptance, and value was adopted. According to Spencer-Cavaliere & Watkinson (2010), this interpretation of inclusion supports the amplification of the voices of persons with disabilities, as inclusion is understood as a “subjective experience [requiring] investigation from the perspective of the child who is ‘to be included’” (p. 275). Thus, throughout this study, the term inclusion is used to describe the subjective experiences described by the participants (Spencer-Cavaliere & Watkinson, 2010) of belonging, acceptance, and value.

Given our conceptualization of inclusion as a subjective experience, it is critical to engage with students with disabilities themselves about their experiences to understand if they viewed PE to be inclusive. In studies that examined the inclusiveness of integrated PE classes from the perspective of persons with disabilities (Spencer-Cavaliere & Watkinson, 2010), participants generally described not feeling ‘included’ despite existing in the same physical space as their peers. As such, this line of inquiry appears to support assertions by Haegele (2019) that “integrated [PE] settings may not be providing inclusive experiences for students with disabilities” (2019, p. 394). Challenging experiences within integrated settings appear to be informed by instances of teasing and limited peer engagement (Spencer-Cavaliere & Watkinson, 2010); feelings of incompetency, low self-esteem, and being ‘on display’ (Haegele, 2019); restricted participation and a lack of appropriate accommodations (Haegele, Kirk et al., 2020).

Based on how students with disabilities reflected on their experiences in integrated PE settings, it appears there is a clear need for practical strategies that can enhance the inclusiveness of these experiences. Of concern though, is that no research exists that explored how students with disabilities experienced PE when these strategies were implemented. By referring to these strategies as ‘best practices’ and promoting their use by physical educators without having this data to support their benefits, it is possible that students with disabilities may be unintentionally harmed, as “a lack of awareness of the complexities of individual experience of disability among policy makers and practitioners can engender practices which, however well intentioned, have the potential for unintended and often un-noticed consequences for the young person being ‘included’” (Atkins, 2016; p. 8). More research is needed that investigates the experiences of students with disabilities in integrated PE settings to understand how these practices were perceived on the inclusiveness of their experiences. That is, research is necessary that examines whether these ‘inclusive’ strategies can help support feelings associated with inclusion (i.e., acceptance, belonging, value) among those with disabilities from their embodied, first-person perspectives. As such, the purpose of this study was to examine how students with orthopedic impairments experience strategies identified in the literature to support ‘inclusion’.

## Method

We conducted this study through a social constructivist lens, with the belief that individuals cultivate subjective meanings of their experiences as they attempt to develop an understanding of the unique contexts within which they live (Creswell, 2014). As such, we sought first to understand the context or setting in which participants exist and then to gather information about their experiences (Creswell, 2014). Our own subjective truths inevitably influenced the data collection and analysis processes as well; therefore, we must first explicitly state our own personal and professional positionalities (Hopkins et al., 2017). To that end, the authors all possess backgrounds in education and education research, with expertise in both qualitative and quantitative inquiries using interviews, observations, and survey methodologies. The first three authors have extensive backgrounds in both PE and adapted PE/physical activity and the fourth has vast experience working with students with orthopedic impairments and has served on several collaborative research teams with an adapted physical education focus. All authors are financially independent and do not identify as members of the disability community; however, two of the authors do have immediate family members who identify as having a disability.

### Research Approach

We used an interpretative phenomenological analysis (IPA) research approach, in alignment with a social constructivist worldview, to examine the PE experiences of students with orthopedic impairments with strategies intended to promote ‘inclusion’. It is important to note that the term ‘orthopedic impairment’ has been used intentionally throughout this manuscript. While this term is often used interchangeably in both the literature and adapted sport contexts with the phrases ‘physical disability’ or ‘physical impairment’, ‘orthopedic impairment’ is specifically used in the United States education system to identify and describe students receiving special education services. To qualify for special education services under this category a student may have any number of disabilities affecting their physical mobility, such as a congenital anomaly, impairment caused by disease (i.e. poliomyelitis), cerebral palsy, spina bifida, or spinal cord injury (IDEA, 2004).

IPA is a qualitative research approach with two central aims. First, researchers attempt to understand the participants’ world and adequately describe specific events from their perspective (Larkin et al., 2006). In this study, the specific events of interest are the participants’ PE experiences. Secondly, researchers must perform an interpretative analysis where they seek to understand and describe the meanings and feelings that participants attribute to the events of interest (Larkin et al., 2006). IPA has roots in phenomenology, hermeneutics, and idiography (Smith et al., 2009) in that it closely examines the lived experiences of the participants from their own unique lens, emphasizes that the researcher is an active participant in a two-stage hermeneutic process, and seeks not to make generalizations about groups or populations, but rather to understand the unique experiences of each individual participant (Smith et al., 2009).

### Participants

To recruit interview participants, the first author sent a recruitment packet with a welcome letter, a description of the research purpose and protocol, and her contact information, to personal contacts via email. Personal contacts included former colleagues (adapted physical education teachers and physical therapists), adapted sport coaches, and parents of youth with orthopedic impairments. She also posted an abbreviated version of the welcome letter on her personal social media accounts to reach a maximum number of potential participants. Both the welcome letter and the social media posts indicated that interested parties should contact the first author directly to obtain more information about the study. She then distributed a consent form, assent form, and demographic questionnaire via email to all individuals who expressed interest. The demographic questionnaire included open-ended questions about the participants’ identities (age, gender, race/ethnicity, disability status) and availability for interviews, as well as closed-ended questions about school experiences (type of school and PE class attended) to determine whether those interested were eligible for participation in the study. Since all potential participants were under the age of 18, the first author communicated with and obtained consent from parents or guardians before speaking with the participants themselves. Once eligibility was determined, a one-on-one meeting was held via video chat to answer any questions that the parents or guardians had prior to obtaining consent. At the end of the meeting, each parent then signed and returned the consent form to the first author via email. When consent was obtained, the first author conducted a one-on-one video call with the participants themselves, reading the assent form aloud and obtaining verbal assent. At this time all potential participants who assented to participate were enrolled in the study. The Institutional Review Board at Old Dominion University reviewed and approved these research protocols.

A sample of six interview participants (aged 10–14 years; four females and two males) was purposively sampled for this study to include those who: (a) were currently enrolled in a K-12 school in the United States, (b) were between the ages of 10 and 14 years old, (c) were currently enrolled in an integrated PE class, (d) self-identified as having an orthopedic impairment as defined by the IDEA (2004), (e) did not have an intellectual disability/IQ of less than 70, and (f) were willing to complete two interviews that were approximately 60- to 90-minutes each. Participation was not limited by specific gender, race/ethnicity, or socioeconomic categories. Three participants identified as White, one as Caucasian, one as Black, and one as Asian. Three participants utilized mobility aids for ambulation, and three ambulated independently without mobility aids. Of those who used mobility aids, one student utilized a manual wheelchair for mobility, one student utilized a power wheelchair for mobility, and one student utilized arm crutches for mobility. All participants attended integrated PE classes in either public (*n* = 5) or private (*n* = 1) K-12 schools. The participant that attended a private school at the time of data collection had been enrolled in public school for his entire education other than the current school year. Given the digital nature of data collection, potential participants were able to join the study from anywhere in the United States, yet all of the recruited participants resided in either the Southeast or Mid-Atlantic regions. For privacy purposes, the location of each individual participant will not be shared. Participants were offered the opportunity to select their own pseudonym for data presentation purposes to increase confidentiality. Three of the six participants elected to choose their own (Ramen Noodle, Agnes, and Alice), and the first author selected the remaining three. These names, as well as additional demographic data can be found in Table 2.

**Table 2:** *Participant Demographics*

Pseudonym	Gender	Age	Grade	Race/ Ethnicity	Orthopedic Impairment	Mobility Aid(s) Used	School Setting
Agnes	Female	13	7th	Black	Bilateral Radioulnar Stenosis & ADHD	None	Public
Alice	Female	11	6th	White	Col6 Muscular Dystrophy	Power Wheelchair	Public
Gordon	Male	10	5th	White	Cerebral Palsy/Hemiplegia	None	Private
Maggie	Female	12	4th	Asian	Spina Bifida	None	Public
Phillip	Male	14	9th	White	Osteogenesis Imperfecta	Manual Wheelchair	Public
Ramen Noodle	Female	10	4th	Caucasian	Above the Knee Amputee & Type 1 Diabetes	Arm Crutches/ Prosthesis	Public

### Data Collection

Data were collected in three ways for this study. First, each participant was sent a written prompt via email. Participants were instructed to write as much or as little as they wanted to answer the question about their experiences. The written prompt read “please describe the degree to which you feel included in your PE classes, as well as any strategies that your PE teachers use that help you to feel more or less included”. Participants were given one week to complete the prompt, which allowed them time to consider their answers and reply with more detail than they might in the interviews. The written prompts were distributed prior to the interviews so that the first author could ask clarifying questions or probe further into the responses if needed during the interviews (Alred et al., 2019). Participants were permitted to handwrite, type, dictate to a scribe, use assistive technology, or audio record their responses, before returning them to the first author via email.

After the responses to the written prompts were returned, the first author and participants identified a day and time that they were both available to engage in one, semi-structured video call interview. Due to technological difficulties, one interview was completed over the phone. Interviews lasted between 23-and 57-minutes, with an average of 39-minutes. Video interviews were selected as the data collection type for this study due to the diverse geographical locations of participants and restrictions preventing in-person interviews related to COVID-19. The interviews for this study followed a semi-structured interview guide with questions that were developed based on strategies described on education websites (Nagro et al., 2016; Wang, 2013; Williston, 2017), articles in PE practitioner journals (Ellis et al., 2009; Lieberman, et al., 2019), and an ‘inclusion’ rating scale for PE (Lieberman, et al., 2017). Based on these resources, 16 specific strategies were selected as target strategies. Information about these 16 strategies can be found in Table 1. Sample interview questions included: (a) how have you felt when your PE teachers made equipment modifications or changes for you in your PE class? and (b) how have you felt about being given choices in the activities you participate in or the modifications you receive in your PE class?

After the initial construction of the interview guide, it was reviewed by a panel of experts, including one adapted PE researcher who primarily conducts qualitative research using the IPA approach, one child with an orthopedic impairment, and one adapted PE teacher. The first author sent each panel member a document outlining the purpose of the study and research questions, along with the interview guide, and asked for feedback as to the relevance and clarity of questions. In total, the panelists recommended editing two questions for clarity. The first author then infused those suggestions into the final draft of the interview guide. The first author began each interview by describing the purpose of the study and her background to expose her positionality before beginning the questions on the interview guide. The guide was then used flexibly throughout the interviews to allow the participant to dictate the magnitude and order of the discussed topics (Smith & Sparkes, 2017), while also serving as a checklist to ensure that the same general topics will be addressed by all participants.

During and after each interview, the first author took reflective interview notes in the margins of the interview guide. These notes represented the third form of data for this study and reflected the researcher’s feelings about the tone of the interview, the rapport between the first author and the participant, topics and/or quotes that stood out as particularly meaningful, and thoughts about potential themes (Smith & Sparks, 2017). During this note taking process, the first author was able to reflect on and identify any possible personal biases that may have affected the interview or the following presentation of the interview data. Finally, the reflective note taking process allowed the first author to conceptually return to the context of the interview when reviewing the data during the analysis process (Walker et al., 2013).

### Data Treatment and Analysis

Each audio recording was transcribed verbatim upon completion of the interviews. The data were then treated using a four-step IPA data analysis procedure (Smith et al., 2009). First, the first author immersed herself in the data by reading and rereading the transcriptions, written prompts, and reflective interview notes multiple times. The purpose of this step was to familiarize herself with the data, which allowed her to make reflective, interpretative notes and comments on the initial emergence of themes. Second, the first author reduced the data into emergent themes by highlighting key phrases and developing meaningful labels with which to code them. During this step, the first author made additional interpretative notes. To aid sense-making, the first author drew on the second author as a critical friend to check and challenge initial emergent themes and to deepen the first author’s engagement and understanding of the participants’ experiences (Tracy, 2013). Third, the first author compared emergent themes within each participants’ documents to form clusters of related themes. Lastly, the first author compiled overall descriptions of themes from patterns and connections detected across the entire participant group. Themes were then reviewed by the first and second authors to ensure coherence within each theme and that the content was reflected by theme titles.

### Quality Assessment

We followed four principles for assessing the quality of qualitative research as presented by Yardley (2000) and recommended by Smith and colleagues (2009) for use in IPA studies: (a) sensitivity to context, (b) commitment and rigor, (c) transparency and coherence, and (d) impact and importance. According to Yardley, sensitivity to context addresses the context of theory and related literature, social and cultural contexts, and the balance of power between the researcher and the interviewee (2000). We addressed these concepts by conducting a thorough review of related literature and selecting an appropriate framework for the study, beginning the interviews with an explicit description of the first author’s positionality, and carefully considering the role of the participant as an expert in every stage of the study’s design. Yardley described commitment as the responsibility of the researchers to have a prolonged engagement with the topic, develop competence in the methods used, and immerse themselves in the relevant data; and rigor as the thoroughness of the data collection and analysis processes (2000). We addressed commitment and rigor by carefully identifying inclusion criteria for the participants that aligned with the research questions and research approach, and by employing appropriate and meaningful data analysis procedures. Transparency and coherence relate to the version of reality that is constructed within the resulting manuscript (Yardley, 2000). We demonstrated a commitment to transparency and coherence by selecting appropriate participants to detail the phenomena (i.e., students with orthopedic impairments themselves rather than stakeholders); explicitly describing the data collection, data treatment, and analysis protocols; explicitly identifying positionality, potential biases, and reflexivity; and presenting verbatim textual representations of the participants’ accounts (Yardley, 2000). The final principle will be determined by those consuming this manuscript, as the impact and importance of qualitative research lies in the authors’ ability to communicate the content as such to the reader (Yardley, 2000).

## Results

Three interrelated themes were constructed based on the data analysis: “It’s kind of embarrassing”: Experiences with support; “I don’t want to be different”: Equipment, activity, and rule modifications; “I like to be a part of the conversation”: Autonomy and choice in PE; and “I would rather be like the other students”: Discussing disability. In each theme, participants described either and/or both positive or negative experiences with teachers who either did or did not implement the suggested strategies. While the participants were questioned about all 16 strategies listed in Table 1, they either had no experience with, or neutral feelings about, half of the strategies. As such, experiences related to arrival/departure instruction, warm-ups, differentiation, demonstrations, fitness testing, and feedback do not appear in the results below. Each of the eight remaining topics evoked salient memories associated with feelings of varying degrees of ‘inclusion’ in participants’ PE classes, and appear throughout this section.

### “It’s Kind of Embarrassing”: Experiences with Support

The engagement of support personnel, in the form of paraprofessionals, teacher aids, and/or adapted PE teachers, in the integrated PE space was among the most common suggestions for ‘inclusive’ strategies in the professional literature (Lieberman, et al., 2017; Williston 2017). This suggestion was supported by Phillip’s experiences, as he reported having adult support provided to him by either an aide or physical therapist who was actively engaged with him throughout PE and helped him to feel safe and included. He shared:

It makes me feel included more to have an aide because they are worried about my safety at all times. When I was younger and my teacher tried harder to include me, my aide only had to monitor some things, because everything was already relatively safe. But as things were getting more dangerous for me when my teachers stopped making modifications to things, they would have to be with me more. Sometimes my physical therapist comes and helps modify things too and that helps because then I don’t have to worry about possibly hurting myself.

It is important to note that Phillip’s perspective and concern over safety may have been unique due in part to his diagnosis of osteogenesis imperfecta or ‘brittle bones’ (reflective notes), as well as the multiple injuries he experienced in PE over the years (reflective notes). Alice had a somewhat different perspective and recalled feeling more included when her aide was *not* actively involved with her throughout her entire PE class. Instead, she felt it benefitted her most when the aide sat off to the side and waited until she requested help. Alice explained how grateful she was to have support that was flexible and allowed her to retain some control:

They do activities with me sometimes and they help me do some of the things I wouldn’t be able to do otherwise. I’m grateful that I have someone who is able to help me and I’m glad that I’m able to be included in it with that way. They sit to the edge until I need help, they wait for me to tell them that I need something. That makes me feel very glad, makes me feel good that I’m able to do and decide that stuff for myself.

Interestingly, while Alice described positive experiences attending PE with her aide, she had strong negative feelings about PE classes that her adapted PE teacher attended. Alice mentioned him several times throughout the interview, each time relating his presence to feelings of embarrassment, discomfort, and decreased value and acceptance (reflective notes). Alice described how:

Coach H makes me feel less included because he has me do other exercises or he doesn’t have me do things right. He doesn’t have me do things similar. He does not treat me similar to everyone else. He treats me like I’m younger than I am. With him I usually do things off to the side or in a different room, but I prefer being in a separate room with him because I would rather not be seen doing something so different from everyone else, especially when he tells me to do something like patty-cake. Though if he is called an adapted PE coach, shouldn’t he be working on making PE more adapted instead of working off on the side with me?

In contrast to Phillip’s positive experience with paraprofessional support staff, Alice’s narratives did not support the use of this ‘inclusive’ strategy. Instead, Alice’s desire to be in a separate room so that her peers would not see her working with her adapted PE teacher was similar to the feelings that Ramen Noodle and Gordon had about paraprofessional support in their PE classes. Gordon shared that “having another adult makes you feel like you’re the center of attention and I don’t like to be the center of attention,” and Ramen Noodle described how when a paraprofessional was supporting her, “I think that other people think I can’t do things by myself, even though I can.” In each of these instances, the participants viewed the support they received from adults as having a negative impact on the perceptions that their peers had of them, hinting that any increased access to the curriculum achieved in using this strategy was not worth the negative influence on feelings of acceptance or belonging (reflective notes).

Ramen Noodle’s disdain for having paraprofessional support during integrated PE influenced her to favor peer buddies, another commonly explicated ‘inclusive’ strategy (Wang, 2013). She shared that:

Adults are for helping other people and it makes me seem like I can’t help myself, and it is kind of embarrassing. I’d rather have a friend or classmate helping because it’s like, ‘oh that’s just a friend helping her out’ and you don’t feel ashamed with that.

Alice, on the other hand, felt it was *more* challenging to solicit help from a peer than an adult, because “a person would rather have your friends see them as able to do things”, hinting at concerns over being accepted by her peers. While their preferences and experiences were in opposition to each other, the underlying feeling driving their perceptions was centered on social capital and peer perceptions rather than access to activities in the PE curriculum (reflective notes). Gordon also felt that having a peer buddy assigned to him was not desirable, but for a slightly different social concern. He did not want his peer to miss out on his own PE experiences, and said that “I don’t like it because then he isn’t doing the exact same thing everyone else is doing because he is helping me.” Ramen Noodle, Alice, and Gordon all seemed unable to separate their own needs from concerns over how these types of ‘inclusive’ strategies might be perceived or experienced by their peers without disabilities (reflective notes). In fact, Phillip was the only participant to mention his own PE experience when discussing peer buddies. Similar to his feelings on adult support, Phillip’s main concern was his safety. He agreed with Alice and Gordon that the implementation of peer supports was not beneficial, but for a different reason. Phillip described that “having a peer buddy isn’t helpful because with an aide there is a lot of medical stuff, so other students don’t really know how to help me.”

### “I Don’t Want to be Different”: Equipment, Activity, and Rule Modifications

Like engagement of support personnel, equipment, activity, and rule modifications proliferate ‘inclusive’ recommendations intended to help enhance participation in PE in the extant literature (Ellis et al., 2009; Nagro et al., 2016). Much like the experiences described in the first theme, participant narratives surrounding the concept of modifications varied, often in direct contrast with one another. Generally, participants felt that meaningful modifications that did not change the nature of an activity promoted feelings of ‘inclusion’, but that inappropriate or nonexistent modifications led to feelings of exclusion or marginalization.

Alice and Phillip both reported having teachers who implemented this strategy, and both felt that it enhanced their feelings of acceptance, belonging, and value, supporting the strategy’s use in integrated PE classes. While whole-group modifications were preferred, Phillip felt more valued and included when his teachers simply made an effort to modify activities, regardless of what the outcome was. He explained that:

Having modifications helps me feel more included, especially when they give the whole class the same modification. I feel good about the changes that they make when I don’t stand out. But I would also be okay if it was just me that had something different because I would know that they were trying to include me.

Alice, however, was a bit more discerning in her approval of modifications. She reported that modifications only helped her to feel more included when she was able to do an activity in a manner similar to her peers, giving her a sense of legitimate participation (reflective notes). She described contrasting experiences with modifications, one being a throwing activity that allowed her to be successful and competitive alongside her peers, and the other being a soccer activity that left her questioning the value of her participation. She described that:

Some changes to activities make me feel more included because I’m doing the same thing. During games when we throw the ball at people, I am allowed to get closer to people in order to hit them. It makes me feel more included partly because it’s fun to be able to hit people with the ball. So, you’re successful, and it’s fun. I like it because it’s a competitive game where you’re really able to win and participate in it. Some other modifications just don’t work really very well. Like in soccer, they put a big plastic thing in front of my chair and the ball would be too small and would catch under my chair. Plus is takes a lot of work to get the big plastic thing on and if you don’t have it just the right way to hit the ball it doesn’t work. If what I’m using is different than everyone else, I’m not really playing the same game anyway, it’s different.

Ramen Noodle and Gordon also reported negative experiences with the utilization of this ‘inclusive’ strategy in their PE classes; however, their perspectives targeted principle rather than about specific modifications they experienced (reflective notes). Ramen Noodle, for example, shared that “I don’t like things that change the activity. If the teacher changed [the activity] I wouldn’t like it and I’d be like ‘oh, this is boring, I want to do what everyone else is doing.” Similarly, Gordon explained how modifications diminished his sense of belonging, sharing that “I feel left out when the teacher changes activities for me. I do not want an advantage. I would rather not win and not have an advantage than have a change made for me.”

Whereas Alice, Ramen Noodle, and Gordon each provided examples of how the utilization of modifications made them feel less included, it is also important to note that a lack of appropriate modifications was also noted as leading to feelings of embarrassment, confusion, and pointlessness. Maggie, for example, wished that her teachers did provide modifications to activities, and felt that she was on display even more when things were not adapted. She wrote:

When things come up that I can’t participate in, I get embarrassed. People want to know why I can’t do it. They stare. I wish I wasn’t in PE on those days and wish I was somewhere else. I don’t want to be different and when I can’t do something or have to do it really differently it makes me embarrassed (written prompt).

Phillip described similar feelings, and described a situation where the lack of modification excluded him from an activity (reflective notes), yet nothing was done to remedy the situation:

During year my PE teacher handed me and my personal aide a jump rope. I was very confused since I utilize a wheelchair daily and jumping rope is impossible. Overall, I have never truly felt included in PE (written prompt).

When asked to elaborate on this experience, Phillip went on to explain that he would have preferred to participate in an alternate activity rather than sitting and watching his peers for the entirety of the jump rope unit. Alice’s experiences echoed this sentiment, as she agreed that in some circumstances, doing something different was favorable to an activity that was simply inaccessible. Alice wrote that:

I personally feel that if it is not something I’m able to do in a similar fashion to my peers I should be given an alternate activity. One activity like that is run day. On run day everyone runs around the field. During that I drive around the field in my chair. There is no point to it since all I am doing is driving around in big circles (written prompt).

More than anything, the participants in this study wanted to feel like valued and legitimate, successful members of their PE classes, and while modifications were one tool utilized in an attempt to promote these feelings, they did not provide a clear solution (reflective notes). Rather, the findings supported modifications as an ‘inclusive’ strategy only when they were implemented meaningfully and with respect to the individual student’s needs and desires (reflective notes). Ramen Noodle’s response to the written prompt seemed to best summarize the collective feelings of the group, as she wrote “I don’t want to sit out! I want to play the games with my friends” (written prompt).

### “I Like to be a Part of the Conversation”: Autonomy and Choice in PE

The concepts of autonomy and choice were discussed with regard to both modifications and establishing teams and partners in PE. The relevant strategies discussed in this theme were those that allowed the students with disabilities to collaborate with their teachers to identify potential modifications (Lieberman, et al., 2019; Nagro et al., 2016), and for the teacher to establish partners and teams for activities rather than allowing students to self-select their groupings (Lieberman, et al., 2017). Overall, the participants supported the use of both of these strategies, with few reporting positive experiences with their implementation and most reporting negative experiences without their implementation.

While modifications were scarce in the recollections of most of the participants (reflective notes), Agnes, Alice, and Maggie all agreed that having some choice in the modifications they used, or in the activities in which they participated, had the potential to increase feelings of inclusion. Agnes shared that “having choice in PE made it better,” and Maggie said that “I like when I have two choices and I get to pick what I want to do.” Alice expanded a bit more on the concept of collaboration with her teacher, saying that “I would rather be asked whether there is another way to do things, I like to be a part of the conversation and help come up with solutions.” Phillip unfortunately, could not recall a time that he was given an opportunity to weigh-in on his experience, but echoed Alice’s desire to collaborate with his teachers. He said that “my teachers in middle school didn’t give me any choices or ask for my input. I would have been comfortable having conversations with them and providing ideas about things that might help instead of just not being included.” The participants in this study seemed primed and ready to advocate for themselves and aid in enhancing their participation in PE, but unfortunately, it appears they were not often given the opportunity to do so (reflective notes).

Student input was also discussed in relation to the establishing of partners and teams in PE, and interestingly, none of the participants recalled having an experience where their teachers implemented the recommended ‘inclusive’ strategy. Instead, Alice, Maggie, and Ramen Noodle described the feelings that they associated with having to find their own partners and groups to work with in PE. While their experiences did vary, the feelings that they described seemed to support the idea that allowing students to choose their own groups was not a beneficial practice. Alice was in favor of selecting her own groups and said that “it helps me to feel included that it’s easy for me to find a partner,” whereas Maggie and Ramen Noodle disagreed. Maggie shared that “it’s kind of hard when I have to pick my own. I don’t know where to go. I like when teachers pick.” Ramen Noodle’s similar feelings about this topic were salient enough that she wrote about them on the written prompt before even engaging with the interview questions, noting “when they are doing something where they are picking people, I am not usually picked. This makes me feel sad that I’m the last one to be picked”. Ramen Noodle then reiterated these feelings of non-acceptance when asked about her experience during the interview, saying that “I don’t get picked as often as everyone else. Makes me feel bummed. Makes me feel less included. I never get picked. I’m always the last one to get picked for a team, makes me feel sad.” A common thread in the first two themes was the participants’ fear of negative peer perceptions, first with support personnel drawing attention to them, and then with concerns about looking different or standing out when participating in activities. In each, participants perceived that their peers might see them as ‘less than’ due to their disability status, a fear that may have been warranted given the data in this current theme (reflective notes). Maggie and Ramen Noodle’s difficulty finding partners and teams in their PE classes, when left to their own devices, suggested that their peers *do* possibly view them as less capable or desirable of a teammate.

### “I Would Rather be Like the Other Students”: Discussing Disability

The final theme addresses the suggestion that teachers should discuss a student’s disability status both with them (Wang, 2013), and with their peers (Williston, 2017). Among the participants, only Ramen Noodle had a conversation about her orthopedic impairment with her teacher at the start of the school year and found it helpful. She explained that:

Okay, well they asked like what I needed and if there was anything that I needed, just come up to them and tell them. And we talked about the stuff that I needed, and it was mostly with my mom and not me. Sometimes they’d ask me one or two questions though and then I’d answer it. I felt like they just wanted to know that if I needed help with anything, just come and ask. I thought it was helpful.

Not only was Ramen Noodle the only participant to have experience with this strategy, but she was also one of only two participants who believed it to be beneficial. Phillip, who largely described feelings of exclusion, an inability to participate, and a lack of control over his experiences in PE, expressed positive feelings toward disability disclosure. Phillip seemed to crave some collaboration and communication with his PE teachers (reflective notes) as he shared that:

None of my teachers have asked me about my disability. I think only one teacher had ever heard of what it was. I would feel more included if they asked me about what I need and how I could participate more.

In contrast, Gordon, Agnes, and Maggie did not support the utilization of this ‘inclusive’ strategy and felt positively about not discussing their disability with their teacher. Maggie was glad that her teachers did not talk to her or ask her questions and said that given the choice, she would want to share the bare minimum amount of information with them (reflective notes). She said that “I do not want to talk to my teachers about having spina bifida. I just want them to know that I can’t do running.” Likewise, Gordon felt relieved that his teachers did not address his orthopedic impairment with him. In his written prompt, Gordon attributed his feelings of inclusion in his PE class to this, and wrote:

I like when teachers don’t talk to me about my disability and just treat me like everyone else. I like that they do not treat me differently because I do not like being singled out. I do not need much assistance in the games we play, and they treat me just like everyone else.

Gordon expanded on this further during his interview, saying that if his teachers did ask, he also gave them as little information as possible, saying “I would not be open to having a conversation with my teachers about my disability. If they asked, I would just tell them ‘my doctor told me to wear this leg brace and arm brace, bye!’” Agnes thought positively about her teacher’s lack of inquiry about her orthopedic impairment and felt more included because they did not seem to know about her disability (reflective notes). Agnes shared that even if they did ask, “I would not tell them. I’m scared that I’m going to be treated differently.” Gordon and Agnes’ concerns about being treated differently were validated by Alice’s account of her own experiences (reflective notes). Her PE teacher approached her with questions about her disability, and she preferred that they had not. She explained that:

I would rather be able to focus on my work instead of answering a bunch of questions. I like to be able to be immersed in my work and I like to be able to do it similar to what everyone else is. I would rather be like the other students and just get on with my day.

Gordon’s and Agnes’ level of comfort with the idea of disability disclosure seemed to track with their narrative responses in the previous themes (reflective notes). They each described not wanting modifications to be made for them. Specifically, Gordon felt most included when he did not have any sort of support personnel and Agnes enjoyed having choice in the activities she participated in during PE. They both appeared comfortable with their level of participation without the implementation of “inclusive” strategies; therefore, they did not see a benefit to discussing their orthopedic impairments with their teachers. Although there was some disparity among the participants regarding whether they wanted to discuss their disability with their teacher, there was one ‘inclusive’ strategy that unanimously elicited negative responses from those who had experience with it.

Gordon, Alice, and Phillip were vehemently opposed to the practice of a teacher discussing disability with students without a person with a disability present (reflective notes). Gordon’s opposition to this idea was even stronger than to the possibility of a one-on-one conversation with his PE teacher (reflective notes), explaining that if a conversation about his disability was *required*, that the information should come from him. He described an experience from years earlier where a teacher discussed his disability with his classmates in his absence Gordon felt embarrassed that the discussion did not provide his classmates an accurate representation of his disability, thus he does not like the idea of it happening again in the future. He said that “I would rather tell people myself, face-to-face. If the teacher doesn’t know everything, she could say something that’s not true.” For Alice and Phillip, being talked about rather than included in the conversation, led to peers treating them differently and lasting experiences of marginalization (reflective notes). Phillip described an experience when:

I’ve had teachers tell my classmates things about my disability when I’m not there, and then everyone treats me differently, like I couldn’t do as much, or they had to be nice to me. They thought they were being helpful, but it just made me mad.

Alice, too, experienced a shift in treatment after her teacher’s seemingly well-intentioned talk with her peers (reflective notes). Like Gordon, Alice stated that if the conversation had to occur, she would rather be involved, sharing that “nobody wants to think that a group of people’s talking about them when they’re not there, and now people give them special treatment and they don’t know why.”

## Discussion

This study examined how students with orthopedic impairments experienced strategies identified in the literature to support ‘inclusion.’ The results of this study aligned with previous findings in that participants experienced restricted participation and a lack of modifications (Haegele, Kirk et al., 2020), felt excluded due to limited peer engagement (Spencer-Cavaliere & Watkinson, 2010), and experienced feelings of being ‘on display’ (Haegele, 2019). This study’s unique contribution to the literature, however, was the discussion of these participants’ experiences with regard to specific ‘inclusive’ strategies implemented by the participants’ PE teachers. Collectively, the participants’ experiences supported several strategies, such as offering student choice with regard to activities and modifications and having teacher generated partners and teams rather than those that were student selected, as being those that could contribute to feelings of acceptance, belonging, and value. Conversely, the participants recalled that other strategies, such as close proximity adult staff supervision and teachers discussing their disability with their peers without their knowledge or input, may be more marginalizing than inclusive.

In this study, we adopted a conceptualization of inclusion that was aligned with a subjective experience associated with feelings of belonging, acceptance, and value. Generally, belonging, acceptance, and value are considered fluid and contextual, with no one individual experiencing them universally at all times (Walker, 1999). As such, a person may experience belonging, acceptance, and value differently in each unique context and phenomena in their life (integrated PE classes, for example). Generally, participants in this study described a lack of belonging, acceptance, and value, regardless of the implementation of ‘inclusive’ strategies. Instead, narratives in each theme portrayed experiences of fear of ‘standing out’ or being ‘othered,’ such as Maggie’s description of feeling watched when unable to perform PE tasks in the same manner as her peers. The participants’ narratives often returned to concern over how capable or desirable their peers perceived them to be, rather than confidence in their place amongst the group, suggesting that feelings of belonging, acceptance, and value were scarce. The findings were consistent with the idea that PE is often an environment where students with disabilities experience negative social interactions with both teachers and peers, making it a class where feelings of belonging and inclusion are unlikely to occur (Holland & Haegele, 2021).

According to Atkins, “the notion that inclusion is something that can be reduced to a set of strategies or inspection criteria is concerning” (2016, p. 8). This assertion is supported by the findings of this study, where the varied experiences that participants had with these ‘inclusive’ strategies suggested that however well intentioned, a one-size-fits all approach to integrated PE is not appropriate, and that blanket suggestions as to how to ‘include’ students cannot, and should not be made (Haegele, Kirk et al., 2020). Further, when ‘inclusive’ strategies are applied universally, there is potential for unintended harm to the person being ‘included’ (Atkins, 2016). Indeed, this inadvertent consequence was seen with the participants in this study. Alice, for example, felt belittled and embarrassed by the modifications and activities that her adapted PE teacher presented to her. Therefore, while this teacher applied an ‘inclusive’ strategy to his work with her, the implementation of the strategy fell short. This teacher’s failure to engage in reciprocal conversations with Alice about her own feelings of success and belonging throughout the instructional process contributed to her feelings of being excluded and reinforced the inequities that she was already facing (Haegele, 2019). Rather than silencing and objectifying students by assigning them to passive roles in their own educational experiences (Shah, 2007), and using cookie-cutter sets of ‘inclusive’ strategies, teachers must instead be reflexive to each individual student’s needs and provide opportunities for students to play an active role in the education process (Davis & Watson, 2001).

Among the strategies discussed with participants, it is important to note that one received universal, fervent opposition; the practice of teachers discussing their disability without them being present. For the participants, this ‘inclusive’ strategy led to marginalizing experiences, where they suddenly felt like they were being treated differently by their peers without knowing the cause. Rather, the participants (e.g., Gordon, Alice) expressed a desire to represent their own realities should a teacher ever feel the need to share information about them with their classmates. Interestingly, the use of disability simulations, an ‘inclusive strategy’ often implemented at the post-secondary level, has received parallel feedback from individuals with disabilities in recent years (Leo & Goodwin, 2016). In both the K-12 and postsecondary versions of this strategy, an adult, presumably without a disability, shares disability information with a class full of students (also without disabilities) in an effort to reduce prejudice and improve attitudes toward individuals with disabilities (Leo & Goodwin, 2016). Instead, the lack of disability representation in these strategies can impose “ableistic norms” and perceptions of inability (Leo & Goodwin, 2016 p. 169) on the part of those with disabilities, resulting in increased negative perceptions on behalf of those without disabilities (Leo & Goodwin, 2013). The literature, in combination with the results from this study, provide support for the recommendation that disability should not be discussed without disability representation present.

## Conclusion

The purpose of this study was to examine how students with orthopedic impairments experience strategies identified in the literature to support ‘inclusion.’ Four themes were constructed which portray varying degrees of feeling ‘included’ based on the specific context surrounding each participants’ experiences. Overall, the themes did not support the blanket use of any suggested ‘inclusive’ strategies, and instead suggested that the use of some strategies should be reconsidered. More specifically, the participants in this study felt marginalized when their teachers discussed their disability status with their peers without their knowledge. Thus, PE teachers should look to collaborate with their students with disabilities to co-construct ‘inclusive’ practices that are appropriate in each unique situation (Haegele, Kirk et al., 2020).

## Data Availability

Due to privacy restrictions, data are not available beyond the direct quotations included within this manuscript.
